# Wearable Biomechanics and Video-Based Trajectory Analysis for Improving Performance in Alpine Skiing

**DOI:** 10.3390/s26031010

**Published:** 2026-02-04

**Authors:** Denisa-Iulia Brus, Dorin-Ioan Cătană

**Affiliations:** 1 Department of Motor Performance, Transilvania University of Brașov, 500036 Brașov, Romania; 2 Department of Materials Engineering and Welding, Transilvania University of Brașov, 500036 Brașov, Romania; catana.dorin@unitbv.ro

**Keywords:** alpine skiing, performance analysis, trajectory deviation, wearable sensors, image processing, polynomial regression, sport informatics, XSensDOT

## Abstract

Performance diagnostics in alpine skiing increasingly rely on integrated biomechanical and kinematic assessments to support technique optimization under real training conditions; however, many existing approaches address trajectory geometry or biomechanical variables separately, limiting their explanatory power. This study evaluates an integrated analysis framework combining OptiPath, an AI-assisted video-based trajectory analysis tool, with XSensDOT wearable inertial sensors to identify technical inefficiencies during giant slalom skiing. Thirty competitive youth athletes (*n* = 30; 14–16 years) performed controlled runs with predefined lateral offsets from the gates, enabling systematic examination of the relationship between spatial trajectory deviations, biomechanical execution, and performance outcomes. Skier trajectories were extracted using computer vision-based methods, while lower-limb kinematics, trunk motion, and tri-axial acceleration were recorded using inertial measurement units. Deviations from mathematically defined ideal trajectories were quantified through regression-based calibration and arc-based modeling. The results show that although OptiPath reliably detected trajectory variations, shorter skiing paths did not consistently produce faster run times. Instead, superior performance was associated with more efficient biomechanical execution, reflected by coordinated trunk–lower limb motion, controlled vertical loading, reduced lateral corrections, and higher forward acceleration, even when longer trajectories were followed. These findings indicate that trajectory geometry alone is insufficient to explain performance outcomes and support the integration of wearable biomechanics with trajectory modeling as a practical, low-cost, and field-deployable tool for alpine skiing performance diagnostics.

## 1. Introduction

Alpine skiing is a sport in which performance is often determined by differences of only milliseconds, meaning that even subtle technical inefficiencies can substantially affect race outcomes. Previous research has shown that skiing performance is strongly influenced by the interaction between trajectory selection, movement coordination, and terrain characteristics, particularly in high-speed, turn-intensive disciplines such as giant slalom [1,2]. However, accurately capturing these performance determinants under real training conditions remains challenging, as many existing assessment methods are either laboratory-bound, technically complex, or insufficiently integrated for practical coaching use [3,4]. The outdoor nature of the sport, large elevation changes, highly variable course topography, and challenges associated with obtaining accurate field-based measurements limit the effectiveness of traditional performance-monitoring methods. These constraints underscore the need for accessible technologies capable of reliably capturing skier trajectories and providing objective, actionable feedback without requiring advanced analytical expertise. The OptiPath application was developed in response to this need, enabling video-based extraction of skiing trajectories, comparison with ideal paths defined by gate configuration, and rapid identification of deviations in real training environments. To maximize usability, the system was designed to rely on commonly available devices such as smartphones, operate independently of specialized IT skills, and remain robust across diverse terrain conditions, despite the positioning inaccuracies inherent to general-purpose equipment.

Advances in motion analysis have highlighted the value of integrating biomechanical and kinematic information into contemporary alpine skiing training methodologies.

In giant slalom skiing, trajectory-related kinematic variables and joint-level biomechanical measures represent complementary performance indicators: trajectory geometry reflects spatial efficiency and tactical line selection relative to gate positioning, while joint kinematics and acceleration patterns capture movement execution, force application, and the ability to maintain speed and stability through successive turns.

The sport requires rapid directional transitions, precise edge control, and optimized posture at high speeds, conditions in which athletes and coaches benefit from data-driven feedback systems. Previous work has explored rapid-feedback frameworks in elite environments [1], as well as structured assessment models for field sports [2]. Therefore, field-deployable tools capable of jointly assessing trajectory characteristics and joint-level movement patterns are increasingly relevant for performance diagnostics and technique feedback in giant slalom skiing. Quantitative modeling approaches have been proposed to objectively evaluate deviations from idealized skiing trajectories and associated performance gaps [3].

To operationalize this biomechanical framework within real training environments, wearable inertial sensors were incorporated to capture lower-limb kinematics, including three-dimensional acceleration and angular displacement. XSensDOT sensors offer a practical balance between measurement accuracy, portability, and minimal interference with athletic performance, making them well suited for outdoor winter-sport contexts where laboratory-based motion capture systems are impractical [4]. Such characteristics make wearable inertial sensors particularly suitable for field-based performance diagnostics in alpine skiing, where ecological validity and minimal disruption of training routines are critical.

Despite the increasing use of artificial intelligence and wearable technologies in sport science, accessible systems that integrate biomechanical sensing with trajectory-based performance analysis in alpine skiing remain limited. Previous studies have demonstrated the potential of AI-driven approaches for performance prediction and injury risk assessment [5], as well as real-time pose estimation techniques applicable to dynamic sports [6]. However, many existing solutions are restricted to elite environments due to high costs, operational complexity, or the need for specialized equipment [7].

Correlating trajectory-based metrics with biomechanical variables such as knee flexion and multidirectional acceleration provides a multidimensional understanding of skiing technique. Prior studies demonstrate that combining spatial and biomechanical analyses improves the interpretation of complex athletic movements and supports the identification of subtle execution errors, fostering structured and progressive training [8]. Recent literature further emphasizes the expanding role of wearable biomechanics in sport performance enhancement and injury prevention [9] and shows how multilayered feedback from wearable systems can enhance motor learning and technical correction in skill-intensive sports [10]. Platforms such as SnowMotion have demonstrated the feasibility of IMU-based real-time feedback in skiing [11], reinforcing the relevance of integrated solutions [12]. Additionally, contemporary perspectives highlight the importance of real-time data visualization in coaching workflows, enabling faster corrective decisions and improved athlete engagement [13].

Despite these advances, important gaps remain in the current literature. Most existing approaches address either biomechanical variables or trajectory geometry in isolation, limiting the ability to explain why performance deviations occur during skiing. Moreover, accessible, low-cost systems that integrate video-based trajectory analysis with wearable inertial sensing in real training environments remain scarce. In particular, the relationship between gate-by-gate trajectory deviations, biomechanical execution patterns, and performance outcomes in giant slalom skiing has not yet been systematically investigated. Addressing these gaps requires performance assessment frameworks that jointly interpret spatial trajectory behavior and biomechanical execution using wearable and video-based systems that are feasible in real training environments.

Accordingly, the present study was designed to address these gaps by testing the hypothesis that integrating video-based trajectory analysis with wearable inertial sensing provides a more comprehensive and informative assessment of alpine skiing performance than trajectory analysis alone. Specifically, it was hypothesized that:

(1) The OptiPath system can reliably quantify deviations from an ideal skiing trajectory during giant slalom skiing;
(2) Greater trajectory deviations are associated with distinct biomechanical patterns, including reduced knee flexion and altered acceleration profiles, as measured using wearable IMU sensors;
(3) Athletes who demonstrate more efficient biomechanical execution may achieve competitive performance outcomes even when skiing longer trajectories.

## 2. Materials and Methods

### 2.1. Study Design and Participants

The experimental setup was designed to replicate realistic alpine skiing conditions while maintaining sufficient control over key parameters. A giant slalom course (GS) containing ten gates was established on a 100 m slope, with approximately 10 m spacing between gates.

Thirty competitive youth athletes (14–16 years), all affiliated with local alpine skiing clubs, participated in the study. Each athlete performed multiple runs along the course while maintaining predetermined lateral offsets of 0.5, 1.0, 1.5, or 2.0 m from each gate. This controlled design enabled systematic assessment of how trajectory deviations influence path length and performance outcomes.

Participants were selected in collaboration with club coaches to ensure technical homogeneity and regular training participation. Athletes with recent injuries or medical restrictions were excluded from the study.

### 2.2. Ethical Considerations

The study was conducted in accordance with the Declaration of Helsinki. According to national regulations and institutional policies applicable at Transilvania University of Brașov, formal ethical approval by an institutional review board was not required, as the procedures involved exclusively non-invasive performance monitoring during regular training sessions and did not include the collection of medical, clinical, or sensitive personal data.

Written informed consent was obtained from the legal guardians of all participants prior to data acquisition, and assent was obtained from the athletes themselves. Authorization to conduct the study and to use training-derived performance data for scientific purposes was provided by the governing bodies of the participating alpine skiing clubs.

### 2.3. Data Acquisition

All runs were recorded using smartphone cameras positioned laterally to the course, employing the manufacturer’s default video recording settings. These settings provided sufficient temporal and spatial resolution for reliable gate detection and trajectory extraction using the OptiPath computer-vision pipeline, a custom in-house software developed by the authors for this study. As the analysis focused on spatial trajectory reconstruction rather than frame-level temporal event detection, the use of standardized default recording parameters did not compromise the accuracy or robustness of the trajectory measurements.

GPS-based tracking was performed using the Ski Track Lite application (version 3.4, Fitness & Sports Apps SRL, Carbonate, Como, Italy), which provided coordinate measurements with three-decimal precision despite an approximate ±4 m positional error. GPS data were used to establish gate coordinates and served as a spatial reference for trajectory analysis [14,15].

Video recordings and inertial sensor data were acquired concurrently during each trial. High-resolution video footage was recorded at a fixed frame rate using smartphone cameras positioned laterally to the course. Xsens DOT sensors (Xsens Technologies B.V., Enschede, The Netherlands) recorded inertial data at a sampling frequency of 60 Hz using second-generation hardware (v2) with firmware version 2.0.0 and Xsens DOT software version 2021.0. Temporal synchronization between video and IMU data streams was achieved by aligning the start of each recording using a common temporal reference, based on the initial gate passage visible in the video and the corresponding acceleration peak detected in the IMU signals. This procedure ensured consistent frame-to-sample alignment across trials and enabled combined spatial–biomechanical analysis.

### 2.4. Data Processing and Modeling

Trajectory analysis was performed using OptiPath, a Python-based desktop application developed for this study. OptiPath integrates computer-vision algorithms with geometric modeling to automatically detect gates and extract skier paths, building on advances in markerless motion capture and pose estimation [16]. These trajectories are then compared with mathematically generated ideal trajectories based on carving equations [17].

To complement spatial data with biomechanical insight, XSensDOT inertial measurement units (IMUs) were mounted bilaterally on the thigh and shin, and in the lumbar region. Sensors recorded tri-axial acceleration and angular displacement at 60 Hz, enabling detailed analysis of segmental kinematics—particularly knee flexion—and trunk behavior during turns. The importance of sensor placement and orientation for IMU accuracy in skiing has been emphasized in the recent literature [18].

**Figure 1:**
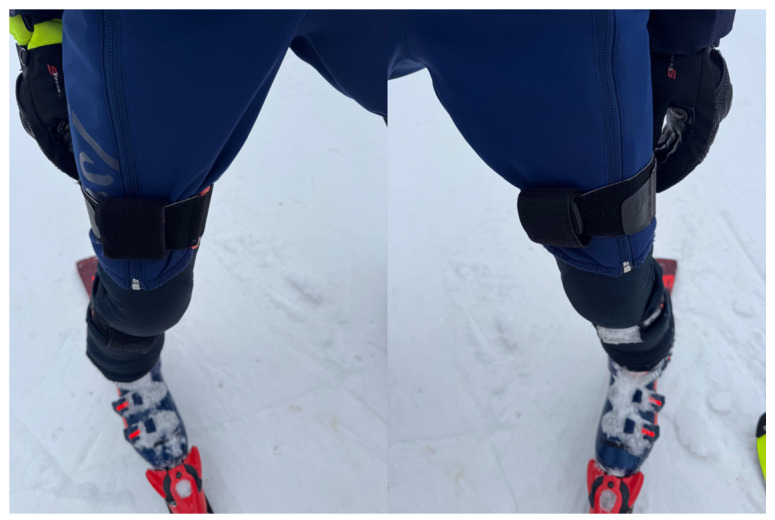
Sensor positioning on the athlete.

Raw inertial sensor data were processed using custom Python scripts (Python version 3.8). Acceleration and angular velocity signals were filtered using a low-pass Butterworth filter to reduce high-frequency noise associated with vibration and sensor movement artifacts (Figure 1). Sensor coordinate systems were transformed into a common anatomical reference frame based on sensor orientation at initialization.

Knee flexion angles were derived from the relative orientation between thigh and shank sensors using joint angle reconstruction based on segmental orientation data. Acceleration components were analyzed along three orthogonal axes corresponding to vertical, lateral, and forward directions relative to the skier’s movement. All biomechanical variables were computed consistently across trials to ensure comparability between subjects and conditions.

Joint angles were expressed using Euler angle representations. To minimize the risk of singularities and discontinuities associated with Euler-based formulations, angle ranges were limited to the sagittal-plane motion relevant for knee flexion during giant slalom skiing, and orientation sequences were selected consistently across all trials.

All collected data—including trajectory deviations, biomechanical variables, and calculated inefficiencies—were compiled into comparative datasets and visual overlays. Microsoft Excel and Python scripts were used for post-processing. Turn radius and angular displacement at each gate were derived from the curvature of the skier’s path. To estimate path length, the following assumptions were made: (1) the skier traveled along a circular arc around each gate, (2) radius and angle of the arc were known for each gate, and (3) each turn was completed using a single circular arc of constant radius. The arc-based path segment was computed using:

tj_i_ = 2 π r_i_α_i_/360 = π r_i_α_i_/180, (1)

where:

tj_i_—extra path for gate i;

r_i_—is the estimated radius of the skier’s path around gate i;

α_i_—is the corresponding angular displacement of gate i.

Polynomial regression was selected to map image-based measurements to real-world spatial coordinates due to its robustness in approximating nonlinear relationships under field conditions, where perspective distortion and camera placement variability are unavoidable. This approach provides a stable transformation while minimizing overfitting within the calibrated distance range.

The arc-based trajectory estimation model was adopted based on established biomechanical and physical representations of carved ski turns, in which the skier’s center of mass follows a quasi-circular path around each gate. The primary assumptions of this model include a constant turn radius per gate and smooth, continuous directional changes, which are consistent with controlled giant slalom skiing. Together, these modeling choices allow the estimation of additional path length and associated time loss in a computationally efficient and biomechanically interpretable manner.

To calibrate OptiPath’s trajectory outputs, seven distinct giant slalom course configurations were set up at different locations, each featuring unique gate layouts and terrain characteristics. Seven athletes completed all configurations, performing three runs at each prescribed lateral distance (0.5, 1.0, 1.5, and 2.0 m). GPS coordinates of all gates were recorded prior to trials. The coordinates and corresponding .mov video files were then uploaded to the application, which generated ideal and actual trajectories for each run. Subsequent analysis quantified how skiing closer to or further from the gate altered total path length.

### 2.5. Statistical Analysis

Descriptive statistics were used to summarize trajectory deviations, knee flexion angles, and acceleration variables across participants and trials. Given the exploratory and methodological nature of the study, no inferential statistical comparisons were performed. All biomechanical and trajectory-related variables were processed and analyzed consistently across subjects to ensure comparability between conditions.

The term “correlation” is used descriptively throughout the Section 3 and Section 4 to denote observed relationships between biomechanical and trajectory-related variables, without implying inferential statistical testing or significance.

**Figure 2:**
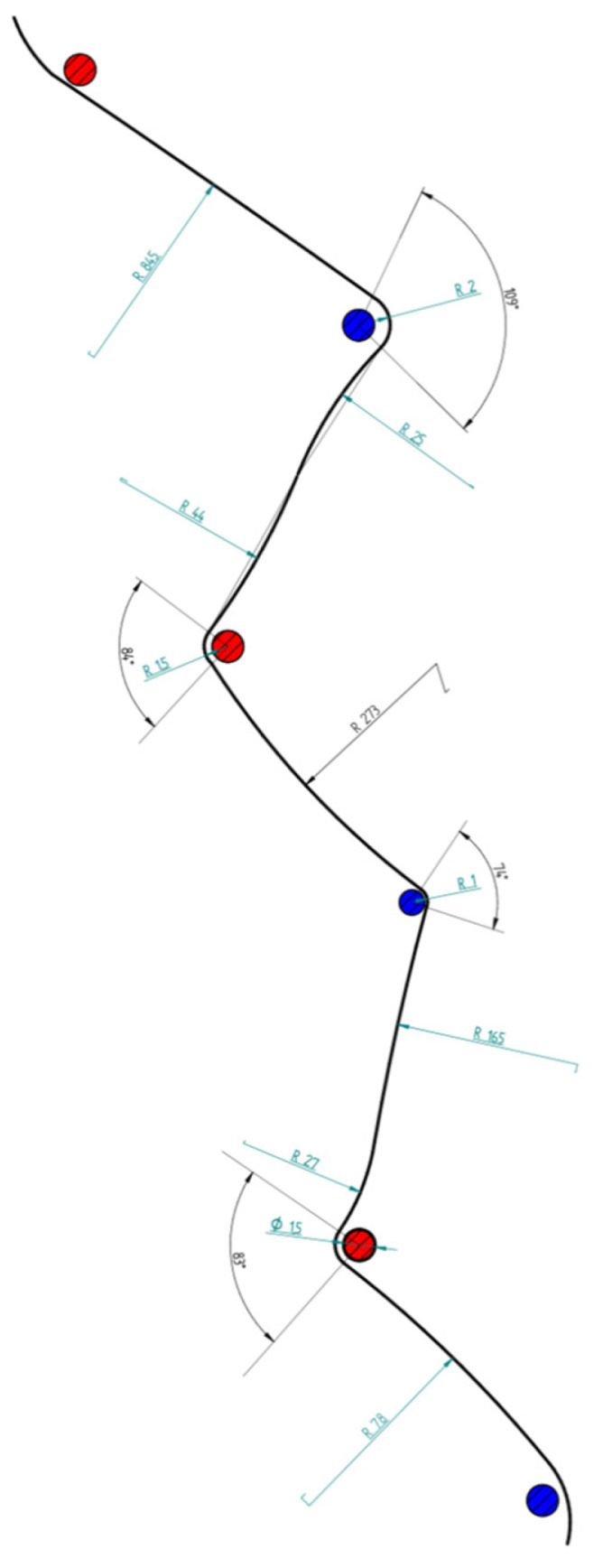
Schematic illustration of the arc-based trajectory modeling approach used to estimate path length deviations in giant slalom skiing, highlighting the geometric parameters turn radius (r) and angular displacement (α).

Figure 2 provides a schematic representation of the geometric assumptions underlying the arc-based trajectory estimation method. The illustration clarifies how turn radius (r) and angular displacement (α) are defined and used in the computation of additional path length around each gate. Quantitative results derived from this modeling approach are presented in Section 3.

**Figure 3:**
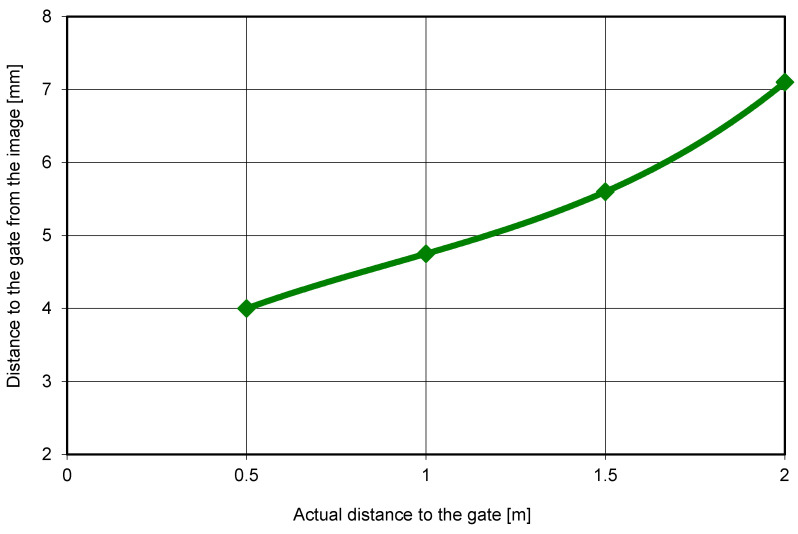
Relationship between the actual distance to the gate and the distance measured in the image.

The data collected during calibration were processed, and knowing the distance at which the athlete passed each gate for every recording, the graph shown in Figure 3 was constructed. Figure 3 illustrates these differences between the course’s representative segments.

## 3. Results

### 3.1. Trajectory Deviations and Modeling Output

**Figure 4:**
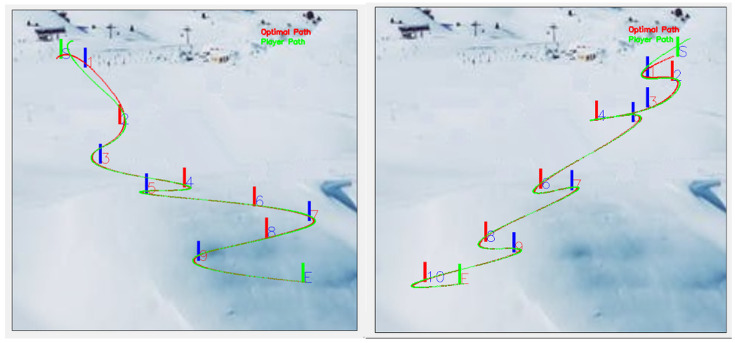
Skier’s ideal and actual trajectory for 2 m (**left**) and 0.5 m (**right**).

**Figure 5:**
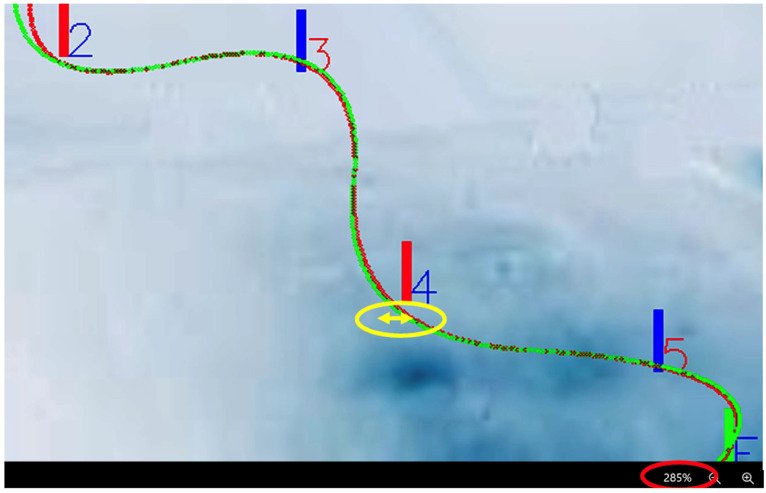
The skier’s ideal and actual path over 2 m (magnification × 285).

The OptiPath system generated detailed visualizations of skier trajectories relative to the mathematically defined ideal line for each course configuration. Clear differences were observed between runs performed at lateral offsets of 0.5 m and 2.0 m, particularly in course sections requiring higher curvature and more pronounced directional changes. As illustrated in Figure 4, athletes skiing closer to the gate followed shorter and more direct trajectories, whereas increased lateral distance resulted in longer, more curved paths. These deviations were especially evident in technically demanding segments, where trajectory elongation became progressively more pronounced with increasing offset. Figure 5 provides a magnified view of representative course sections, further emphasizing the relationship between lateral deviation and measurable path elongation.

**Figure 6:**
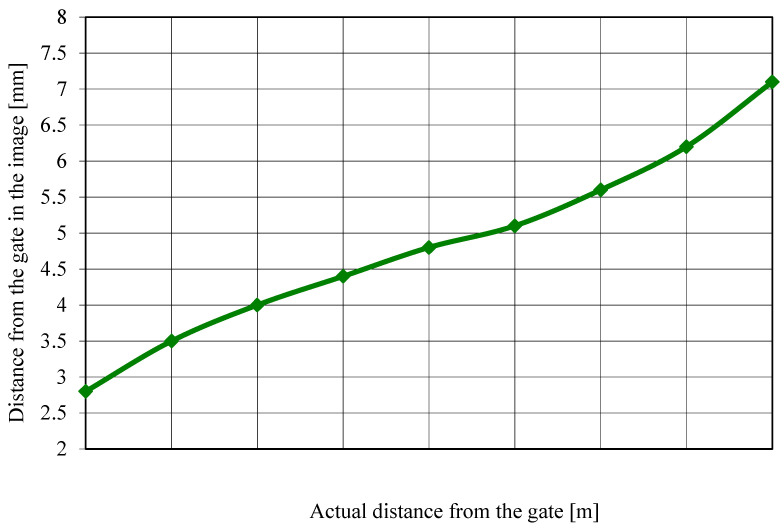
Relationship between the actual distance from the gate and the distance measured in the image, established using a polynomial calibration model.

The polynomial regression function described in Section 2 was applied to convert pixel-based image measurements into real-world lateral distances. This transformation enabled automated, frame-by-frame estimation of each skier’s offset from the gate throughout the run. As shown in Figure 6, the relationship between the actual gate-passing distance and the image-based measurement demonstrated high agreement across the calibrated rang of 0–2 m. These results validate the reliability of the regression model and support its use for automated lateral deviation estimation within the OptiPath framework.

Table 1 summarizes the pixel distances extracted by OptiPath alongside their corresponding real-world distances for all tested offsets (0.5 m, 1.0 m, 1.5 m, and 2.0 m). The consistency of these paired values across repeated trials demonstrates the robustness of the image-to-distance conversion process. Minimal variability between measurements further confirms the stability and repeatability of the regression-based transformation.

### 3.2. Time Loss and Gate-by-Gate Distance Analysis

Using the arc-based modeling approach described previously, the additional distance accumulated at each gate was quantified for all participants. Table 2 presents individual overshoot values by comparing the actual path length with the personalized ideal trajectory derived from each athlete’s geometry and measured gate offset. Across the cohort, accumulated deviations over the full course reached values of up to approximately 20 m relative to the individualized ideal trajectory.

Beyond spatial deviations, the model translated these geometric differences into estimated time loss. While athletes exhibiting larger lateral deviations generally accumulated longer trajectory distances, completion times did not follow a strictly linear relationship with path length. For instance, Subject 4 recorded a total real trajectory of 24.25 m yet achieved a completion time of 22.62 s, outperforming several athletes who followed shorter paths. These findings indicate that race time is influenced by additional biomechanical factors that interact with geometric efficiency and cannot be explained by trajectory length alone.

### 3.3. Biomechanical Patterns Captured by XSensDOT Sensors

The XSensDOT inertial sensors mounted on the thighs, shins, and lumbar region enabled detailed assessment of joint kinematics and whole-body acceleration patterns throughout the giant slalom runs. Analysis focused on two primary biomechanical variables:

- knee flexion angles, reflecting lower-limb compression during turns;
- tri-axial acceleration, with X representing vertical, Y lateral, and Z forward acceleration.

**Figure 7:**
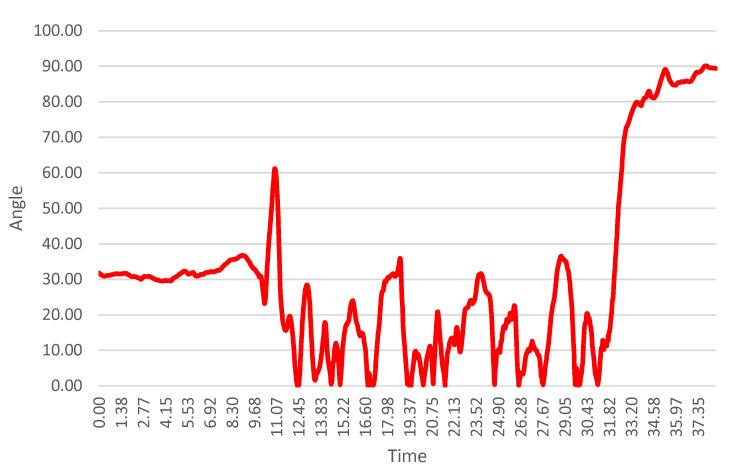
Knee flexion angle time series for the left lower limb of Subject S1, derived from filtered IMU orientation data. Signals were low-pass filtered to reduce high-frequency noise, and the displayed profile represents smoothed joint kinematics across the entire run.

**Figure 8:**
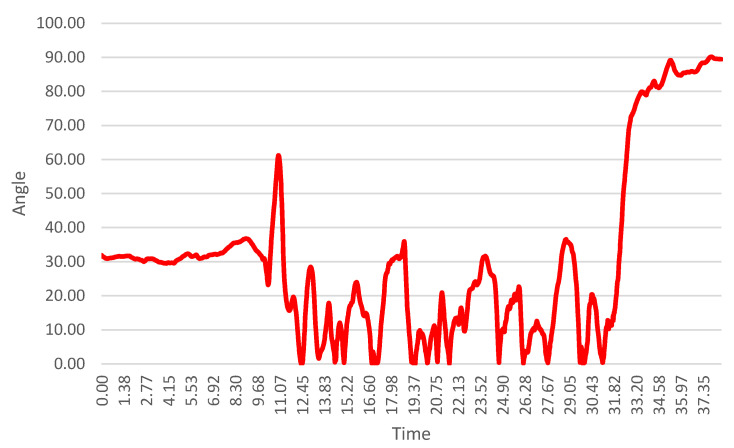
Knee flexion angle time series for the right lower limb of Subject S1, computed from processed IMU data following noise filtering and sensor alignment, allowing qualitative interpretation of flexion patterns during turning phases.

Figure 7 and Figure 8 present knee flexion angle profiles for the left and right lower limbs of Subject S1. Across the entire cohort, knee flexion rarely exceeded 60°, even during high-demand turning phases. This limited flexion range suggests a relatively elevated center of mass and a reduced capacity for leg absorption, both of which may negatively affect carving efficiency, stability, and ski–snow interaction.

The knee flexion angle time series shown in Figure 7 and Figure 8 were derived from processed IMU orientation data and do not represent raw sensor outputs. Prior to angle computation, inertial signals were low-pass filtered to reduce high-frequency noise and movement artifacts. The resulting profiles, therefore, reflect smoothed, continuous joint kinematics across the entire run. Turn phases were interpreted qualitatively by relating the temporal evolution of the knee flexion signal to gate-passage timing observed in the synchronized video recordings.

Acceleration data revealed distinct patterns differentiating efficient and inefficient runs. Athletes who demonstrated high forward acceleration (*Z*-axis), combined with controlled vertical loading (*X*-axis) and minimal lateral corrections (*Y*-axis), tended to produce smoother, more stable trajectories. In contrast, subjects exhibiting elevated lateral acceleration values showed more erratic directional changes, reduced energy transfer efficiency, and increased overall path length.

**Figure 9:**
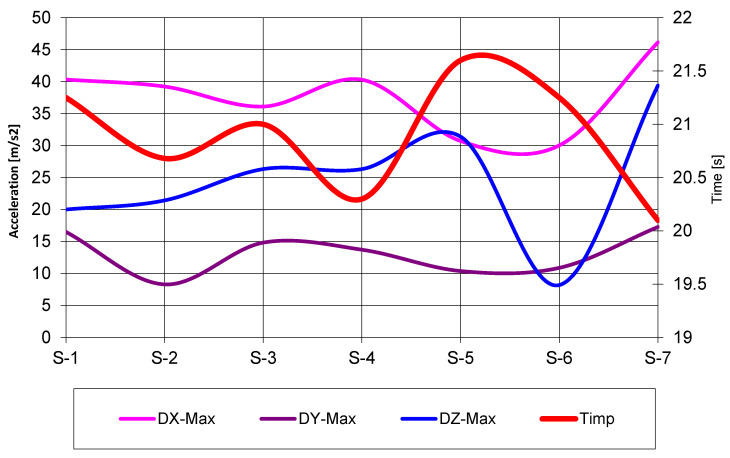
Evolution of skiers’ accelerations for both lower limbs (*X*, *Y* and *Z* directions; minimum, maximum, and average acceleration).

Figure 9 illustrates aggregated acceleration values for both lower limbs, including minimum, maximum, and mean values across all axes. These results further support the relationship between acceleration signatures and total run time, highlighting that movement quality and biomechanical coordination play a decisive role in performance outcomes beyond purely geometric considerations.

The acceleration data shown in Figure 9 represent aggregated values derived from multiple runs for each subject. Acceleration components were expressed relative to the skier’s movement direction and analyzed along vertical (*X*), lateral (*Y*) and forward (*Z*) axes. For each axis, minimum, maximum, and mean values were computed over the duration of the run. To ensure comparability across subjects and trials, acceleration magnitudes were evaluated using a consistent sensor placement and identical data processing procedures. The presented values, therefore, summarize representative biomechanical loading patterns rather than isolated events or single time points.

## 4. Discussion

The findings of the present study demonstrate that alpine skiing performance emerges from the interaction between spatial trajectory characteristics and biomechanical execution, confirming the relevance of jointly analyzing path kinematics and joint-level movement patterns as sport-specific performance indicators. While trajectory deviation represents an intuitive and quantifiable determinant of performance, the results indicate that athletes who followed longer paths did not necessarily record inferior race times. Instead, performance outcomes were strongly influenced by kinematic patterns, force application strategies, and coordination between the trunk and lower limbs. This observation is consistent with previous studies indicating that alpine skiing performance emerges from the interaction between trajectory selection and movement execution rather than from geometric optimization alone [8,14].

Trajectory analysis performed using the OptiPath system showed a high level of consistency and relative accuracy in identifying deviations from the mathematically defined ideal line under controlled training conditions. This capability allows coaches and analysts to localize inefficiencies at specific gates or course segments with precision. However, as evidenced by the results, geometric information alone does not fully explain performance variability among athletes. Several subjects maintained competitive times despite substantial deviations from the ideal trajectory, suggesting the presence of compensatory biomechanical strategies that mitigate geometric inefficiencies.

Although knee flexion angles provided valuable insight into lower limb movement patterns, their interpretation should be considered in light of the applied signal processing approach. The angles discussed in this study were derived from filtered inertial sensor orientation data and represent smoothed joint kinematics rather than raw measurements. While this processing enhances signal stability and interpretability under field conditions, it may attenuate short-duration peaks associated with rapid absorption or high-frequency movement components. Consequently, the reported knee flexion values should be interpreted as representative movement trends rather than exact instantaneous joint extremes. Future work incorporating higher sampling rates, additional sensor fusion strategies, or complementary optical validation could further strengthen joint angle accuracy. Similar considerations regarding the interpretation of IMU-derived joint kinematics under field conditions have been reported in previous biomechanical studies on alpine skiing and other dynamic sports [4,14].

Although the sensor configuration allowed estimation of relative orientation between the thigh and lumbar segments, hip joint angles were not included in the present kinematic model. This decision was motivated by the increased uncertainty associated with hip angle estimation using limited IMU configurations under dynamic field conditions, where soft tissue artifacts and sensor alignment errors may compromise joint-level accuracy. Consequently, the analysis focused on knee flexion and trunk motion as more robust and reliable indicators of lower-limb function and whole-body coordination during giant slalom skiing. Future studies may extend this framework by incorporating refined hip joint modeling through additional sensors or complementary validation methods.

The biomechanical data captured by the XSensDOT sensors were essential for identifying these compensatory mechanisms. Across participants, knee flexion values were generally limited, rarely exceeding 60°, indicating insufficient lowering of the center of mass during turning phases. Such limitations can reduce effective ski–snow interaction and edge engagement. Nevertheless, athletes who achieved superior performance often compensated through alternative strategies, including more efficient trunk rotation, precise weight transfer, and dynamic forward projection. In this context, data from the lumbar sensor proved particularly informative, providing insight into trunk behavior and upper–lower body coordination—factors that play a critical role in maintaining balance, stability, and directional control but are frequently underestimated in conventional performance assessments. The importance of trunk coordination and upper–lower body synchronization for balance and directional control in alpine skiing has been previously highlighted in biomechanical analyses of skilled and elite skiers [7,14].

Acceleration patterns further clarified the relationship between movement quality and performance. Athletes characterized by high forward acceleration combined with controlled vertical loading generally exhibited smoother turn execution and reduced reliance on corrective movements. Conversely, elevated lateral acceleration values were associated with unstable turns, irregular direction changes, and increased trajectory elongation. These observations are consistent with previous biomechanical findings showing that efficient energy transfer and controlled acceleration patterns are associated with improved skiing performance and reduced corrective movements [7,14].

Taken together, these results emphasize that the interaction between trajectory geometry and biomechanical execution is central to alpine skiing performance. Athletes who closely adhered to an ideal geometric line but lacked sufficient knee flexion, trunk coordination, or forward propulsion frequently underperformed relative to those who followed longer trajectories but demonstrated superior neuromuscular control. This finding reinforces the multidimensional nature of skiing performance and highlights the limitations of relying solely on trajectory-based metrics for technical evaluation.

The integrated OptiPath–XSensDOT system offers several practical advantages for performance analysis and coaching. First, it provides objective, frame-by-frame geometric tracking that enables rapid identification of inefficient skiing lines. Second, it supplies detailed segmental kinematic information that reveals the biomechanical causes underlying observed deviations. Third, it supports individualized technical feedback by accounting for athlete-specific biomechanical signatures rather than applying generic correction models. Finally, its portability, low cost, and suitability for outdoor environments make it well adapted to real-world training scenarios.

It is also important to consider the influence of terrain variability and snow conditions on both trajectory behavior and inertial sensor measurements. Variations in slope inclination, surface hardness, and snow friction can affect ski–snow interaction, turn radius, and load transmission through the lower limbs. These external factors may contribute to changes in trajectory geometry as well as to fluctuations in measured acceleration and joint kinematics. Although the present study was conducted under controlled training conditions, such environmental variability is inherent to alpine skiing and represents a relevant contextual factor when interpreting field-based biomechanical data. Accounting for these influences is essential for transferring analytical findings into practical coaching applications. Similar effects of terrain variability and snow conditions on kinematic and inertial measurements have been reported in field-based analyses of alpine skiing [14].

By combining spatial and biomechanical data within a unified framework, this approach supports evidence-based coaching, facilitates monitoring of technical development over time, and may also assist in return-to-sport decision-making following injury by providing objective indicators of movement quality and coordination.

### Study Limitations

Despite the strengths of the proposed integrated analysis framework, several limitations should be acknowledged. First, the study sample consisted exclusively of youth athletes (14–16 years), which limits the generalizability of the findings to older or elite skier populations. Second, although wearable inertial sensors provide practical field-based biomechanical measurements, IMU-derived joint kinematics are subject to sensor drift, soft tissue artifacts, and orientation uncertainties, particularly under dynamic outdoor skiing conditions. Third, trajectory estimation relied on video-based reconstruction and GPS-referenced gate positioning, which may introduce spatial inaccuracies related to camera perspective, terrain variability, and the limited precision of consumer-grade GPS devices. Additionally, environmental factors such as snow hardness, surface friction, and slope inclination could not be fully standardized and may have influenced both movement execution and sensor measurements. Finally, given the exploratory and methodological focus of the study, inferential statistical testing was not performed, and the observed relationships should therefore be interpreted descriptively. Future research should address these limitations by including broader athlete populations, implementing advanced sensor fusion strategies, and employing higher-resolution positioning systems.

## 5. Conclusions

This study demonstrates that integrating computer vision-based trajectory modeling with wearable inertial sensing provides a robust and multidimensional framework for performance diagnostics in alpine skiing. The OptiPath system enables objective quantification of skier trajectories and path-related deviations, while XSensDOT inertial sensors add essential biomechanical context by capturing joint kinematics, trunk coordination, and multi-axis acceleration patterns. Together, these complementary tools allow observable performance outcomes to be directly linked to their underlying biomechanical execution.

The findings confirm that alpine skiing performance cannot be interpreted solely through geometric indicators such as trajectory length. Although skiing closer to the ideal line reduces path length, shorter trajectories did not consistently result in faster run times. Instead, superior performance was associated with more efficient biomechanical execution, characterized by higher forward acceleration, reduced lateral corrections, and effective coordination between the trunk and lower limbs. These results highlight the multidimensional nature of skiing performance and the limitations of trajectory-based metrics when used in isolation.

By combining spatial and biomechanical information within a unified, field-deployable framework, the proposed OptiPath–XSensDOT approach supports evidence-based coaching and individualized technical feedback in real training environments. Future work should focus on expanding validation to broader athlete populations, refining biomechanical modeling, and enhancing spatial accuracy through advanced positioning or multi-camera solutions.

## Figures and Tables

**Table 1:** Calibration relationship between the distance estimated using the OptiPath application and the real distance to the gate.

Distance from the Application [mm]	Real Distance to the Gate [m]
1.00	0
1.50	0
2.00	0
2.50	0
3.00	0.06
3.50	0.25
4.00	0.50
4.50	0.80
5.00	1.20
5.50	1.50
6.00	1.70
6.50	1.80
7.00	2.00
7.50	2.10
9.80	2.50

**Table 2:** Comparison between the distance traveled by the skier and their individualized ideal distance *.

Subject	Path	P 1	P 2	P 3	P 4	P 5	P 6	P 7	P 8	P 9	Finish	Distance Completed (m)	Ideal Distance (m)	Difference	Time (s)
	Angle	50	85	135	170	190	0	155	0	154	0				
**Subject 1**	Real	0.70	1.78	1.88	4.45	0.83	0.00	3.25	0.00	3.23	0.00	16.11			23.55
	Ideal	0.70	0.37	0.00	2.37	0.00	0.00	0.68	0.00	0.67	0.00		4.79	11.32	
**Subject 2**	Real	0.70	1.78	1.18	4.45	1.66	0.00	4.06	0.00	2.15	0.00	15.97			26.43
	Ideal	0.70	0.37	0.00	2.37	0.00	0.00	1.35	0.00	0.67	0.00		5.47	10.51	
**Subject 3**	Real	0.70	0.74	1.18	4.45	1.66	0.00	1.35	0.00	2.15	0.00	12.23			28.64
	Ideal	0.70	0.74	0.00	1.48	0.00	0.00	1.35	0.00	0.67	0.00		4.95	7.28	
**Subject 4**	Real	1.05	2.52	1.18	5.04	6.63	0.00	4.60	0.00	3.23	0.00	24.25			22.62
	Ideal	0.44	0.15	0.00	3.56	1.66	0.00	0.27	0.00	0.27	0.00		6.34	17.91	
**Subject 5**	Real	0.44	1.78	2.83	5.04	5.64	0.00	3.25	0.00	4.57	0.00	23.54			23.02
	Ideal	0.44	0.74	0.24	1.48	0.33	0.00	1.35	0.00	1.34	0.00		5.93	17.62	

* Partial dataset shown for clarity. The full dataset is provided in Supplementary Table S1.

## Data Availability

The data presented in this study are available on request from the corresponding author. The data are not publicly available due to privacy restrictions involving minor participants and the policies of the participating sports clubs.

## Supplementary Materials

The following supporting information can be downloaded at https://www.mdpi.com/article/10.3390/s26031010/s1: Table S1: Correlation between the distance traveled by the skier and their personal ideal distance.
