# Supplementary material for: Wearable Biomechanics and Video-Based Trajectory Analysis for Improving Performance in Alpine Skiing
Source: Sensors (Basel). 2026 Feb 4;26(3):1010. doi: 10.3390/s26031010 (PMC12900127; doi:10.3390/s26031010)
Supplement: Supplementary file 1 [file sensors-26-01010-s001.zip › sensors-4079010-supplementary.pdf]

Table S1: Correlation between the distance traveled by the skier and their personal ideal distance.

Subject	Gate / Path	P 1	P 2	P 3	P 4	P 5	P 6	P 7	P 8	P 9	Finish	Distance completed (m)	Ideal distance (m)	Difference	Time (sec)
	**Angle**	**50**	**85**	**135**	**170**	**190**	**0**	**155**	**0**	**154**	**0**				
**Subject1**	Real	0,70	1,78	1,88	4,45	0,83	0,00	3,25	0,00	3,23	0,00	16,11			23,55
	Ideal	0,70	0,37	0,00	2,37	0,00	0,00	0,68	0,00	0,67	0,00		4,79	11,32	
**Subject2**	Real	0,70	1,78	1,18	4,45	1,66	0,00	4,06	0,00	2,15	0,00	15,97			26,43
	Ideal	0,70	0,37	0,00	2,37	0,00	0,00	1,35	0,00	0,67	0,00		5,47	10,51	
**Subject3**	Real	0,70	0,74	1,18	4,45	1,66	0,00	1,35	0,00	2,15	0,00	12,23			28,64
	Ideal	0,70	0,74	0,00	1,48	0,00	0,00	1,35	0,00	0,67	0,00		4,95	7,28	
**Subject4**	Real	1,05	2,52	1,18	5,04	6,63	0,00	4,60	0,00	3,23	0,00	24,25			22,62
	Ideal	0,44	0,15	0,00	3,56	1,66	0,00	0,27	0,00	0,27	0,00		6,34	17,91	
**Subject5**	Real	0,44	1,78	2,83	5,04	5,64	0,00	3,25	0,00	4,57	0,00	23,54			23,02
	Ideal	0,44	0,74	0,24	1,48	0,33	0,00	1,35	0,00	1,34	0,00		5,93	17,62	
**Subject6**	Real	1,05	1,78	1,18	5,04	1,66	0,00	5,41	0,00	3,23	0,00	19,34			24,22
	Ideal	1,05	0,74	0,00	3,56	0,00	0,00	1,35	0,00	1,34	0,00		8,05	11,30	
**Subject7**	Real	0,44	1,78	1,18	5,04	0,33	0,00	4,60	0,00	3,23	0,00	16,59			23,89
	Ideal	1,05	0,74	0,24	3,56	0,00	0,00	1,35	0,00	0,27	0,00		7,21	9,39	
**Subject8**	Real	0,44	1,78	1,18	3,56	0,33	0,00	5,41	0,00	3,23	0,00	15,92			25,76
	Ideal	1,05	0,74	0,24	1,48	0,00	0,00	3,25	0,00	1,34	0,00		8,10	7,82	
**Subject9**	Real	0,44	1,78	1,18	3,56	3,98	0,00	4,60	0,00	3,23	0,00	18,76			27,66
	Ideal	0,44	0,74	0,00	1,48	0,33	0,00	1,35	0,00	1,34	0,00		5,69	13,07	
**Subject10**	Real	1,05	1,78	0,24	5,04	1,66	0,00	4,60	0,00	3,23	0,00	17,59			23,75
	Ideal	1,05	0,74	0,00	1,48	0,00	0,00	3,25	0,00	1,34	0,00		7,86	9,73	
**Subject11**	Real	0,44	1,78	1,18	5,04	0,33	0,00	4,60	0,00	3,23	0,00	16,59			23,79
	Ideal	1,05	0,74	0,00	1,48	0,00	0,00	3,25	0,00	0,27	0,00		6,79	9,81	
**Subject12**	Real	0,44	1,78	2,83	5,04	1,66	0,00	4,60	0,00	4,57	0,00	20,91			27,21
	Ideal	0,44	0,37	0,00	2,37	0,33	0,00	1,35	0,00	1,34	0,00		6,21	14,71	

**Subject13**	Real	0,44	2,52	1,18	5,04	0,00	0,00	3,25	0,00	3,23	0,00	15,65			27,77
	Ideal	1,05	0,74	0,00	3,56	0,00	0,00	1,35	0,00	0,27	0,00		6,97	8,68	
**Subject14**	Real	0,44	1,78	1,18	3,56	1,66	0,00	3,25	0,00	3,23	0,00	15,08			24,82
	Ideal	1,05	0,74	0,24	3,56	0,00	0,00	1,35	0,00	0,27	0,00		7,21	7,88	
**Subject15**	Real	0,44	1,78	1,18	5,04	3,98	0,00	3,25	0,00	3,23	0,00	18,89			27,14
	Ideal	1,05	0,74	0,00	1,48	0,00	0,00	1,35	0,00	0,27	0,00		4,89	14,00	
**Subject16**	Real	0,70	2,23	0,59	3,56	1,66	0,00	4,06	0,00	3,23	0,00	16,01			27,92
	Ideal	1,05	0,74	0,00	1,48	0,00	0,00	1,35	0,00	0,27	0,00		4,89	11,12	
**Subject17**	Real	0,70	1,78	1,18	3,56	1,66	0,00	4,60	0,00	3,23	0,00	16,70			27,43
	Ideal	1,05	0,74	0,00	1,48	0,00	0,00	1,35	0,00	0,67	0,00		5,30	11,40	
**Subject18**	Real	0,70	1,78	1,88	3,56	1,66	0,00	3,25	0,00	2,15	0,00	14,98			27,65
	Ideal	1,05	0,74	0,00	1,48	0,00	0,00	0,68	0,00	0,27	0,00		4,22	10,76	
**Subject19**	Real	0,70	1,78	1,18	5,04	0,33	0,00	3,25	0,00	2,15	0,00	14,43			27,56
	Ideal	0,44	0,74	0,00	1,48	0,00	0,00	1,35	0,00	0,67	0,00		4,69	9,74	
**Subject20**	Real	0,44	1,78	1,18	3,56	0,33	0,00	3,25	0,00	2,15	0,00	12,68			28,02
	Ideal	0,70	0,74	0,00	1,48	0,00	0,00	1,35	0,00	0,67	0,00		4,95	7,74	
**Subject21**	Real	0,44	1,78	1,18	3,56	0,33	0,00	3,25	0,00	3,23	0,00	13,76			23,91
	Ideal	1,05	0,74	0,00	0,74	0,00	0,00	0,27	0,00	0,67	0,00		3,47	10,29	
**Subject22**	Real	0,70	1,78	1,88	5,04	0,33	0,00	3,25	0,00	3,23	0,00	16,21			27,66
	Ideal	1,05	0,74	0,00	3,56	1,66	0,00	3,25	0,00	0,67	0,00		10,93	5,28	
**Subject23**	Real	0,44	1,78	1,18	3,56	0,33	0,00	3,25	0,00	2,15	0,00	12,68			23,91
	Ideal	0,44	0,37	0,00	1,48	0,00	0,00	1,35	0,00	0,67	0,00		4,32	8,37	
**Subject24**	Real	0,70	1,78	1,18	4,45	0,00	0,00	3,25	0,00	3,23	0,00	14,58			27,13
	Ideal	1,05	0,37	0,00	1,48	0,00	0,00	1,35	0,00	0,67	0,00		4,93	9,65	
**Subject25**	Real	0,70	1,78	0,59	3,56	0,33	0,00	4,60	0,00	3,23	0,00	14,78			27,32
	Ideal	1,05	0,37	0,00	2,37	0,00	0,00	1,35	0,00	0,67	0,00		5,82	8,97	
**Subject26**	Real	0,44	1,78	1,88	4,45	0,83	0,00	3,25	0,00	2,15	0,00	14,78			23,72
	Ideal	0,70	0,74	0,00	0,74	0,00	0,00	1,35	0,00	0,67	0,00		4,21	10,57	
**Subject27**	Real	0,70	1,78	1,88	4,45	0,83	0,00	4,06	0,00	2,15	0,00	15,85			25,34

	Ideal	0,70	0,37	0,00	1,48	0,00	0,00	1,35	0,00	0,67	0,00		4,58	11,27	
**Subject28**	Real	0,44	1,78	1,88	4,45	0,83	0,00	3,25	0,00	3,23	0,00	15,85			24,63
	Ideal	0,70	0,37	0,00	1,48	0,00	0,00	1,35	0,00	0,67	0,00		4,58	11,28	
**Subject29**	Real	0,70	1,78	1,18	3,56	1,66	0,00	3,25	0,00	2,15	0,00	14,27			26,21
	Ideal	0,70	0,37	0,00	2,37	1,66	0,00	1,35	0,00	0,67	0,00		7,13	7,15	
**Subject30**	Real	0,70	1,78	1,18	3,56	0,83	0,00	4,06	0,00	3,23	0,00	15,33			22,46
	Ideal	1,05	0,74	0,00	1,48	0,00	0,00	0,68	0,00	0,67	0,00		4,62	10,71	
